# Nanomaterial-assisted immunodiagnostic profiling and therapeutic targeting of hepatocellular carcinoma: from molecular biomarkers to clinical applications

**DOI:** 10.3389/fimmu.2025.1668630

**Published:** 2025-10-14

**Authors:** Jiping Luo, Jianzeng Ye, Kaipeng Huang, Ziyu Cheng, Liming Liu, Xianpeng Li

**Affiliations:** ^1^ Department of Pathology, Shenzhen Third People’s Hospital (The Second Affiliated Hospital of Southern University of Science and Technology), Shenzhen, Guangdong, China; ^2^ Department of Medical Oncology, Shenzhen People’s Hospital, The Second Clinical Medical College, Jinan University, The First Affiliated Hospital, Southern University of Science and Technology, Shenzhen, Guangdong, China; ^3^ School of Life Sciences, University of Toronto, Toronto, ON, Canada; ^4^ Department of Liver and Infectious Diseases, The Affiliated Lihuili Hospital of Ningbo University, Ningbo, China

**Keywords:** hepatocellular carcinoma (HCC), nanomaterial-based diagnostics, immunogenic epitopes, multi-omics integration, biosensor development, B-cell epitopes, biomarkers, nanodiagnostics

## Abstract

**Aims and objectives:**

This study aimed to identify immunologically relevant transcriptomic and proteomic biomarkers in hepatocellular carcinoma (HCC) and to characterize their B-cell epitopes for potential integration into nanomaterial-based biosensors and immunomodulatory platforms for early diagnosis and targeted therapy.

**Methods:**

We conducted a comprehensive multi-omics analysis by integrating transcriptomic (TCGA-LIHC) and proteomic data to identify differentially expressed genes (DEGs) in HCC. Protein–protein interaction networks and pathway enrichment were used to prioritize hub genes. Five candidate biomarkers, RFC2, HSP90AB1, YWHAZ, CYP2E1, and ADH4, were selected for qRT-PCR and serum ELISA validation in clinical cohorts comprising 85 HCC patients and 50 healthy controls. B-cell epitope prediction was performed using BepiPred 2.0 and validated through synthetic peptide-based ELISA in the same cohort to assess immunoreactivity. Diagnostic performance was evaluated using ROC curve analysis.

**Results:**

RFC2, HSP90AB1, and YWHAZ were significantly upregulated (|log2FC|>0.2) and showed high serological expression, whereas CYP2E1 and ADH4 were consistently downregulated. Predicted B-cell epitopes from RFC2, HSP90AB1, and YWHAZ exhibited strong immunoreactivity (AUC>0.84), indicating their diagnostic potential. Enrichment analysis revealed that upregulated DEGs were involved in cell cycle and mitotic progression, while downregulated genes were linked to immune suppression and metabolic dysfunction. These validated immunogenic epitopes offer promising anchors for nanomaterial-functionalized biosensors, such as gold nanoparticle-conjugated ELISA, graphene-based electrochemical platforms, and peptide-coated quantum dots, for ultrasensitive and multiplexed HCC detection.

**Conclusion:**

By integrating transcriptomic and proteomic screening with epitope-level validation, we identified a novel panel of immunogenic biomarkers suitable for nanomaterial-enabled diagnostics in HCC. These findings support the translational potential of peptide-nano scaffold conjugates in developing minimally invasive, immune-responsive biosensing and therapeutic tools tailored for early-stage liver cancer management.

## 1 Introduction

Cancer remains the leading cause of morbidity and mortality worldwide, with nearly 20 million new cases reported in 2022. Liver cancer alone accounted for 7.8% of cases and was responsible for 830,000 deaths, according to global cancer statistics (1). The intrinsic biological complexity and heterogeneity of cancer present significant challenges to early detection, prognostic assessment, and therapeutic intervention (2). The survival rates for many malignancies remain suboptimal, often due to late-stage diagnosis and the development of therapy-resistant disease. Therefore, the identification of reliable and sensitive molecular biomarkers is critical for early diagnosis, disease progression monitoring, and predicting therapeutic responses (3). Hepatocellular carcinoma (HCC), the predominant form of liver cancer, exemplifies these challenges. As one of the most prevalent and aggressive malignancies, HCC carries a particularly poor prognosis. This is primarily attributable to frequent late-stage diagnosis, the absence of reliable early biomarkers, and constrained therapeutic options (4). Recently, advances in molecular technologies have significantly improved our understanding of tumor biology, but clinical translation remains hindered by the lack of robust, early-diagnosis biomarkers. Recent studies suggest that targeting immune modulation within the tumor microenvironment (TME) could provide therapeutic opportunities, especially in cancers like HCC, where immune evasion mechanisms play a crucial role in tumor progression (5). High-throughput genomic technologies, including transcriptome profiling via microarrays and RNA sequencing, have revolutionized cancer research. These techniques provide comprehensive insights into the molecular landscape of tumors, facilitating the identification of differentially expressed genes (DEGs) that are crucial for tumor initiation, progression, metastasis, and immune evasion (6). Some studies have highlighted the role of immune-related genes in cancer progression and the tumor’s ability to evade immune surveillance, making them attractive candidates for both diagnostic and therapeutic applications (7). In the context of HCC, immune checkpoint molecules, such as PD-1/PD-L1, and tumor-infiltrating lymphocytes (TILs) have been implicated as significant factors influencing prognosis and response to immunotherapy (8). Gene Ontology (GO) and Kyoto Encyclopedia of Genes and Genomes (KEGG) pathway enrichment analyses are commonly used to categorize DEGs based on their biological roles, molecular functions, and involvement in cellular processes. These bioinformatics tools help elucidate the functional relevance of dysregulated genes and provide insight into the pathways that are altered in cancer (9). Specifically, pathways related to immune response, cell proliferation, apoptosis, and metabolic regulation are disrupted in HCC, highlighting potential targets for immunotherapy and precision medicine (10). For instance, the overexpression of immune-related genes such as CD274 (PD-L1) and HLA-G has been linked to immune evasion in HCC, rendering these molecules potential targets for novel immunotherapeutic strategies (11). In recent years, nanotechnology has emerged as a promising approach for improving cancer diagnostics and therapeutics. Nanomaterials, due to their unique size-dependent properties, can be engineered to enhance the delivery and efficacy of diagnostic probes and therapeutic agents, as well as modulate immune responses within the TME. Nanoparticles can be conjugated with specific antibodies or biomarkers, allowing for precise tumor targeting and enhanced imaging (12, 13). Additionally, the use of nanomaterials in drug delivery systems has the potential to overcome drug resistance, a common challenge in HCC treatment (14). Furthermore, nanomaterial-based immune modulation, such as the use of nanoparticles to deliver immune checkpoint inhibitors, has shown promise in preclinical studies, offering new avenues for treating tumors that are resistant to conventional therapies (15). The goal of this study is to explore the molecular mechanisms underlying HCC through an integrative approach that combines high-throughput transcriptomic data analysis with the application of nanomaterials in immunodiagnostics and immunomodulation. Specifically, we aimed to identify immune-related biomarkers for HCC diagnosis and prognosis by conducting differential gene expression analysis and functional enrichment of key genes involved in immune regulation, metabolic pathways, and stress responses. Furthermore, we employed protein–protein interaction (PPI) network analysis to identify hub genes and molecular drivers of tumorigenesis. This investigation also integrates experimental validation through quantitative real-time PCR (qRT-PCR) and enzyme-linked immunosorbent assay (ELISA) for the selected biomarkers. Given the emerging role of immune checkpoints and immune-related pathways in cancer therapy, we also focused on identifying potential targets for nanomaterial-assisted immunomodulation. Our aim is to provide new insights into the molecular basis of tumorigenesis in HCC, leading to the discovery of novel biomarkers for early diagnosis, risk stratification, and personalized treatment. By integrating computational predictions with experimental validation, this study aims to contribute to the development of immune-based diagnostic tools and therapeutic strategies for HCC.

## 2 Methods

This study was approved by the Institutional Research Ethics Committee of The Affiliated Lihuili Hospital of Ningbo University. All experimental protocols were conducted in accordance with the ethical standards of the Declaration of Helsinki.

### 2.1 Transcriptomic analysis

#### 2.1.1 Data acquisition and preprocessing

Microarray gene expression data (CEL files) were retrieved from the NCBI Gene Expression Omnibus (GEO) database (https://www.ncbi.nlm.nih.gov/geo/), comprising paired tumor and adjacent non-tumor liver tissues from hepatocellular carcinoma (HCC) patients. Corresponding platform annotation files were downloaded to map probe identifiers to gene symbols. Raw CEL files were normalized using the Robust Multi-array Average (RMA) algorithm (https://bioconductor.org/packages/affy/) implemented via the *affy* and *limma* R packages (https://bioconductor.org/packages/limma/) available through the Bioconductor platform (https://www.bioconductor.org). Probes with minimal expression across all samples were filtered to reduce noise.

#### 2.1.2 Differential gene expression analysis

A linear model was fitted using the *limma* package, and empirical Bayes moderation was applied to obtain differentially expressed genes (DEGs). Genes with |log2 fold change| > 0.2 and FDR-adjusted p-value < 0.05 were considered significantly upregulated or downregulated.

#### 2.1.3 Heatmap visualization and annotation

A heatmap was generated to visualize the top 100 differentially expressed genes based on adjusted *p*-value and log^2^ fold change. Expression values were z-score normalized per gene across samples. Hierarchical clustering was applied to both rows (genes) and columns (samples). Tumor and non-tumor samples formed distinct clusters. This visualization confirmed robust expression differences between sample groups. Probe identifiers of differentially expressed genes were annotated using platform-specific annotation tables to map them to: Gene symbols and Gene titles. Separate annotated tables were generated for upregulated and downregulated genes.

#### 2.1.4 Functional enrichment and protein–protein interaction network analysis

Functional enrichment analysis of differentially expressed genes (DEGs) was performed using g:Profiler (https://biit.cs.ut.ee/gprofiler) and Enrichr via the GSEApy Python wrapper (https://github.com/zqfang/GSEApy). Annotations were retrieved for Gene Ontology (GO) categories, biological process, molecular function, and cellular component, as well as for KEGG pathways (https://www.genome.jp/kegg/), Reactome pathways (https://reactome.org/), and Human Protein Atlas (HPA) terms (https://www.proteinatlas.org/). Only terms with a false discovery rate (FDR) < 0.05 were considered statistically significant. To explore interaction networks, upregulated and downregulated gene lists were independently submitted to the STRING database (https://string-db.org/) using the following settings: organism Homo sapiens, minimum interaction score of 0.4 (medium confidence), and output limited to input query genes (no additional interactors). Resulting PPI networks revealed central hub proteins and putative protein complexes among DEGs. All transcriptomic analyses, including DEG identification, volcano plots, heatmaps, enrichment terms, and STRING-based PPI networks, were implemented in Google Colab (https://colab.research.google.com/) using a combination of Python 3 and integrated R-based scripts. Annotated DEG tables and associated graphical outputs were compiled and included as **Supplementary Materials** to support reproducibility and interpretation.2.2 Experimental Validation.

#### 2.1.5 Quantitative real-time PCR

To validate the transcriptomic expression profiles derived from microarray-based differential gene expression analysis, quantitative real-time PCR (qRT-PCR) was performed for a subset of top-ranked upregulated and downregulated genes. Tumor and adjacent non-tumorous liver tissue samples were obtained from histologically confirmed HCC patients undergoing surgical resection at The Affiliated Lihuili Hospital of Ningbo University. All samples were immediately snap-frozen in liquid nitrogen and stored at −80°C until RNA extraction. Total RNA was isolated using TRIzol reagent (Invitrogen, USA) following the manufacturer’s instructions, and RNA quality and quantity were assessed using a Nanodrop spectrophotometer (Thermo Fisher Scientific, USA), with integrity confirmed via agarose gel electrophoresis. One microgram of high-quality RNA was reverse transcribed into complementary DNA (cDNA) using the High-Capacity cDNA Reverse Transcription Kit (Applied Biosystems, USA) in a 20 μL reaction volume with the following thermal conditions: 25°C for 10 minutes, 37°C for 120 minutes, and 85°C for 5 minutes to terminate the reaction. Five representative DEGs, RFC2, HSP90AB1, YWHAZ (upregulated), and CYP2E1, ADH4 (downregulated), were selected based on statistical significance (adjusted p < 0.05), biological relevance, and network centrality in STRING analysis. Gene-specific primers were designed using NCBI Primer-BLAST, synthesized commercially (Eurofins, India), and validated for specificity using NCBI BLAST. qRT-PCR was conducted on a StepOnePlus™ Real-Time PCR System (Applied Biosystems, USA) using SYBR Green PCR Master Mix (Applied Biosystems), with each 20 µL reaction containing 10 µL SYBR Green mix, 0.5 µM of each primer, and 2 µL of cDNA template. The cycling conditions included an initial denaturation at 95°C for 10 minutes, followed by 40 cycles of 95°C for 15 seconds and 60°C for 1 minute, and a final melt curve analysis to confirm amplification specificity. GAPDH was used as an internal control for normalization, and relative gene expression was calculated using the 2^−ΔΔCt method. All reactions were performed in triplicate, and data were analyzed using GraphPad Prism (version X), with gene expression differences between tumor and non-tumor tissues assessed by unpaired two-tailed Student’s t-test. A *p* < 0.05 was considered statistically significant.

### 2.2 Enzyme-linked immunosorbent assay

To validate the differential gene expression at the protein level, ELISA assays were performed using serum samples collected from 30 histologically confirmed HCC patients and 30 age- and sex-matched healthy controls (16). Peripheral venous blood was drawn into plain vacutainers, allowed to clot at room temperature for 30 minutes, and centrifuged at 3000 rpm for 10 minutes to separate serum. Aliquots were stored at −80°C until analysis. The selected proteins—RFC2 (UniProt Accession P35250), HSP90AB1 (UniProt Accession P08238), YWHAZ (UniProt Accession P63104), CYP2E1(UniProt P05181), and ADH4 (UniProt Q9QYY9) were prioritized based on significant transcriptomic dysregulation and biological relevance from pathway enrichment and network analyses. Human-specific sandwich ELISA kits were used according to the manufacturers’ protocols: RFC2 (MyBioSource, MBS7200244), HSP90AB1 (Cloud-Clone Corp., SEA415Hu), YWHAZ (CUSABIO, CSB-EL026293HU), CYP2E1 (FineTest, EH2052), and ADH4 (Cloud-Clone Corp., SEA526Hu). Briefly, 96-well plates pre-coated with capture antibodies were used to quantify serum protein levels. Samples were diluted (typically 1:100–1:1000) and assayed in duplicate, alongside standard curves prepared with known concentrations of each analyte. Following incubation at 37°C for 1–2 h, plates were washed thoroughly (4–5 times), and biotin-labelled detection antibodies were applied, followed by HRP-conjugated streptavidin. After final washing, TMB substrate was added and allowed to develop in the dark. The reaction was stopped with 1 N sulfuric acid, and absorbance was read at 450 nm using a microplate reader. Protein concentrations were interpolated from standard curves and expressed as ng/mL or pg/mL. Statistical comparisons between tumor and control groups were performed using unpaired two-tailed Student’s t-tests, with significance set at p < 0.05. Results are presented as mean ± SD, and bar plots display group means with standard deviation error bars.

### 2.3 Epitope identification and peptide synthesis

#### 2.3.1 B-cell epitope prediction

This was conducted using the BepiPred tool IEDB (17). Full-length amino acid sequences of the upregulated proteins RFC2, HSP90AB1 (HSP90-beta), and YWHAZ (14-3–3 protein zeta/delta) were retrieved from UniProt (**Supplementary data 1**). The retrieved sequences were subjected to BepiPred with a default threshold (0.5). The top-scoring epitopes were shortlisted based on their predicted surface accessibility, antigenicity, and length, which was preferably 12–30 aa residues.

#### 2.3.2 Peptide synthesis

All three peptide epitopes were synthesized via solid-phase peptide synthesis (SPPS) using Fmoc (9-fluorenylmethoxycarbonyl) chemistry at a commercial peptide synthesis facility (GenScript, Thermo Fisher Scientific, or equivalent). To enhance stability and better mimic the native conformation of the epitopes, N-terminal acetylation and C-terminal amidation were incorporated during synthesis. The synthesized peptides underwent comprehensive characterization to confirm purity, molecular identity, and structural integrity. High-performance liquid chromatography (HPLC) was performed on a C18 reverse-phase column under a gradient elution system of acetonitrile and water containing 0.1% trifluoroacetic acid (TFA) for purity profiling. Electrospray ionization mass spectrometry (ESI-MS) verified molecular weights by matching observed masses to theoretical values. Additionally, circular dichroism (CD) spectroscopy was conducted in phosphate-buffered saline (PBS, pH 7.4) to evaluate the secondary structural features of the peptides, ensuring reproducibility under physiological conditions.

#### 2.3.3 ELISA-based diagnostic validation

ELISA-based diagnostic validation was performed using serum samples from 30 clinically confirmed hepatocellular carcinoma (HCC) patients and 30 age- and sex-matched healthy controls, as described previously. Peptides (10 µg/mL) were coated in duplicate onto 96-well Nunc MaxiSorp ELISA plates using carbonate-bicarbonate buffer (pH 9.6) and incubated overnight at 4°C. Following coating, plates were blocked with 5% bovine serum albumin (BSA) in PBS containing 0.05% Tween-20 (PBS-T) for 1 hour at 37°C to minimize nonspecific binding. After washing, diluted human serum samples (typically 1:100 in PBS-T) were added to the wells and incubated for 1 hour at 37°C. Plates were then washed, and HRP-conjugated anti-human IgG secondary antibody (diluted 1:5000) was applied, followed by incubation for 1 hour at room temperature. Colorimetric detection was carried out using 3,3′,5,5′-tetramethylbenzidine (TMB) substrate, and the reaction was stopped with 1N sulfuric acid. Data distribution was assessed for normality using the Shapiro–Wilk test. Since the data were normally distributed, statistical comparisons of optical density (OD) values between patient and control groups were performed using an unpaired two-tailed Student’s t-test. Receiver operating characteristic (ROC) curve analysis was conducted to assess the diagnostic sensitivity, specificity, and area under the curve (AUC) for each peptide epitope.

## 3 Results

### 3.1 Differential gene expression analysis

**Figure 1:**
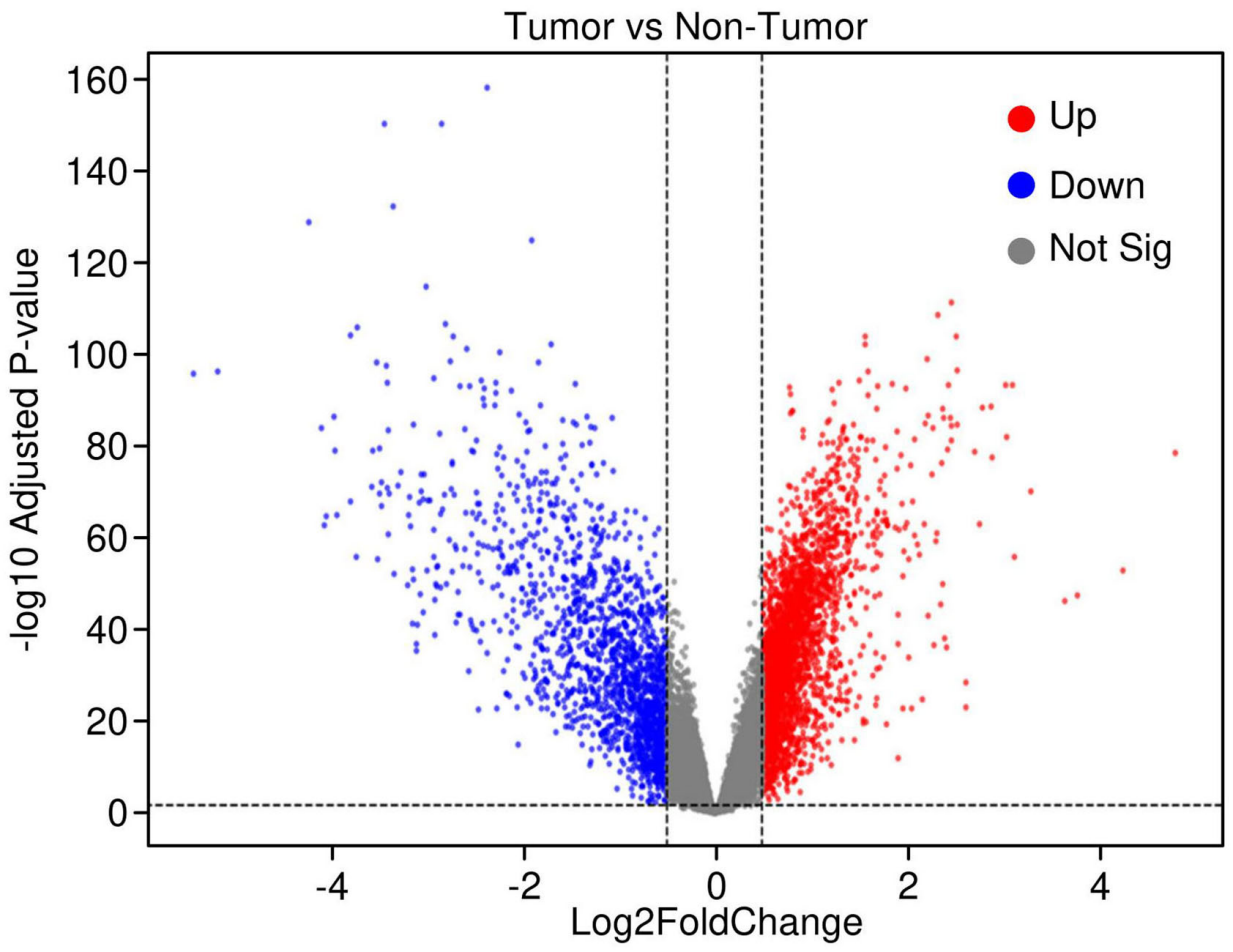
Volcano plot highlighting differentially expressed genes in hepatocellular carcinoma (HCC). Significant genes were identified using thresholds of log_2_ fold change > 0.2 and adjusted *p*-value < 0.05. Upregulated genes are shown in red, downregulated genes in blue, and non-significant genes in grey, illustrating the transcriptomic shifts distinguishing tumor from adjacent normal liver tissues.

Gene expression profiling of 225 hepatocellular carcinoma (HCC) tumor samples and 220 matched non-tumor liver tissues (GSE14520, platform GPL3921) identified significant transcriptional alterations in cancer. After normalization and applying thresholds of |log2 fold change| > 0.2 and adjusted p-value < 0.05, a total of 429 genes were significantly upregulated, while 438 genes were downregulated in tumor tissues. The volcano plot (**Figure 1**) illustrates the distribution of these differentially expressed genes (DEGs), with clear demarcation between significantly up- and downregulated genes.

### 3.2 Heatmap visualization of top DEGs

**Figure 2:**
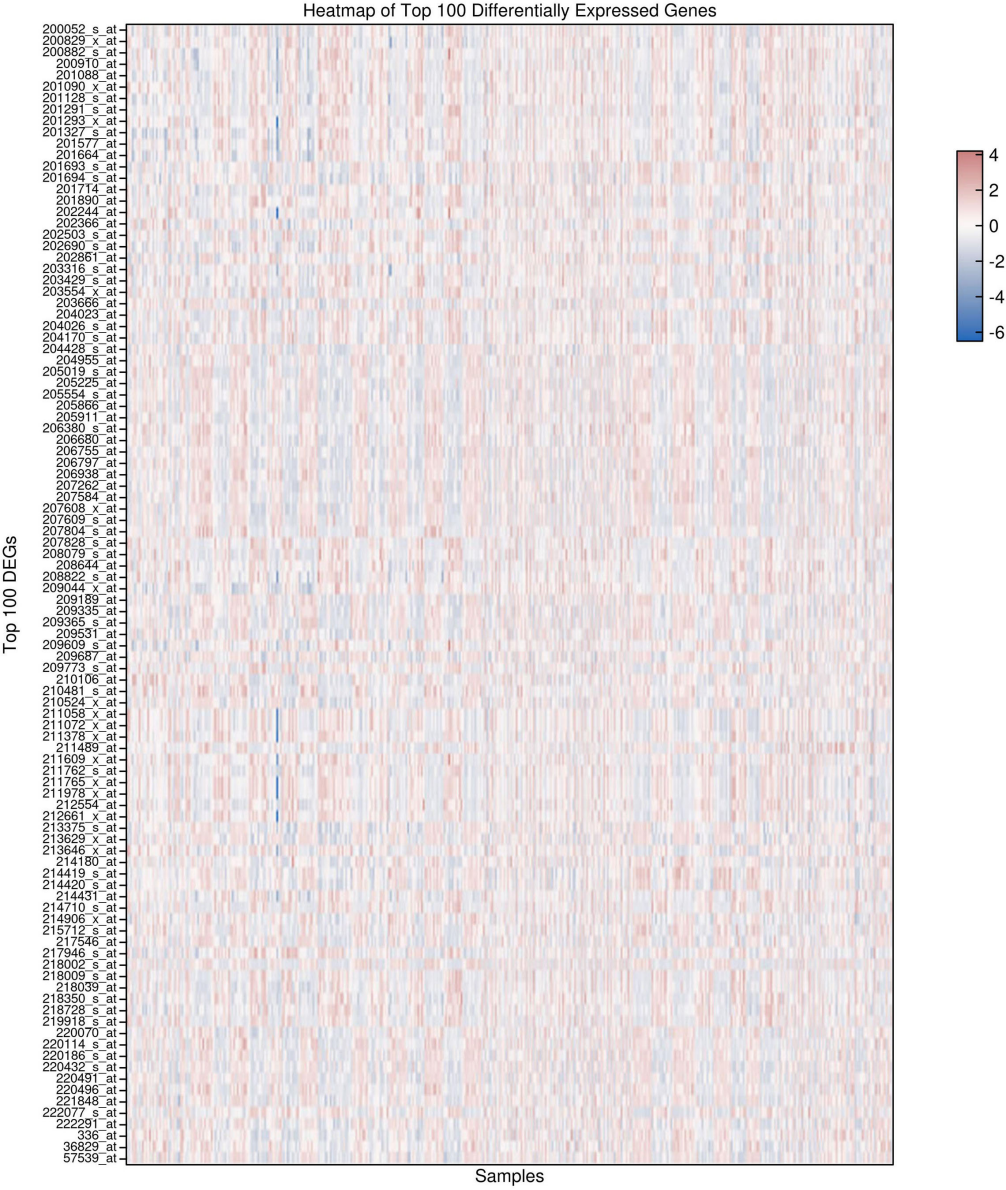
Heatmap of the Top 100 differentially expressed genes in hepatocellular carcinoma (HCC) and adjacent normal tissues. normalized expression profiles demonstrate distinct clustering of tumor versus non-tumor samples, highlighting robust transcriptional differences associated with hepatocellular carcinoma.

The heatmap visualization of the top 100 differentially expressed genes (DEGs), selected based on adjusted p-values, demonstrates a pronounced segregation of expression patterns between HCC tumor samples and adjacent non-tumor liver tissues. Each row represents a specific gene probe, while columns correspond to individual patient samples. Expression values were z-score normalized per gene to facilitate comparison across samples. Hierarchical clustering was applied both to genes and samples using Euclidean distance and complete linkage, resulting in distinct dendrogram branches that separate tumor from non-tumor groups. This stratification highlights a robust transcriptional signature underlying HCC pathogenesis, with clusters of genes exhibiting coordinated upregulation (represented by warmer red tones) or downregulation (cooler blue tones) predominantly in tumor tissues (**Figure 2**). The clear dichotomy in expression profiles further validates the differential gene expression analysis and supports the biological relevance of these candidate biomarkers in distinguishing malignant from normal hepatic tissue states.

### 3.3 Functional enrichment analysis of differentially expressed genes

**Figure 3:**
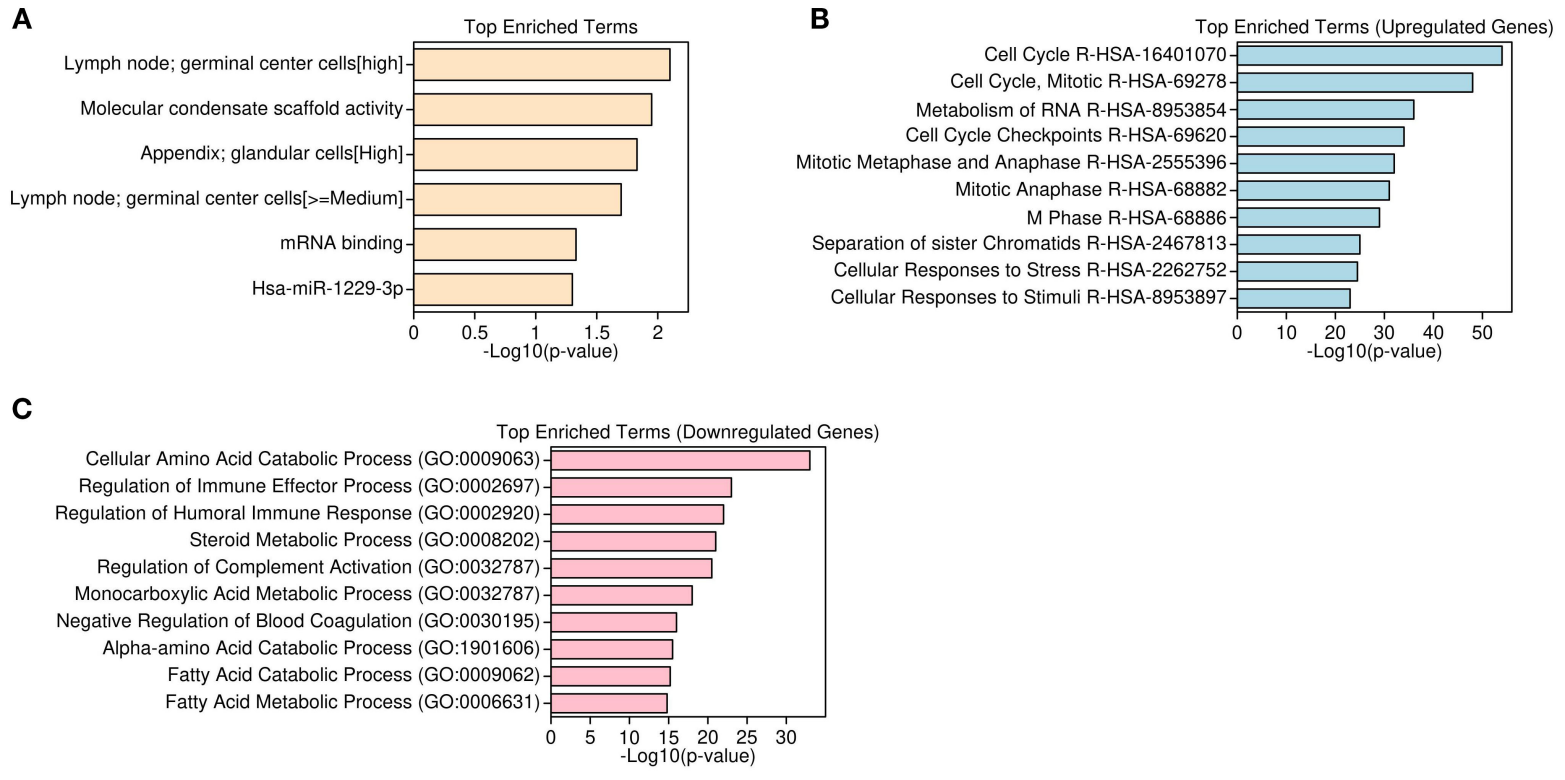
Functional enrichment of DEGs Using GO, HPA, and reactome pathways. **(A)** Top enriched Gene Ontology (GO) and Human Protein Atlas (HPA) terms for upregulated genes. **(B)** Reactome pathway enrichment showing mitosis and RNA processing in upregulated genes. **(C)** GO Biological Processes enriched in downregulated genes, including immune and metabolic functions.

Functional enrichment analysis of differentially expressed genes revealed contrasting biological themes underlying tumorigenesis in HCC. Upregulated genes were predominantly associated with cell cycle regulation, mitotic progression, and RNA metabolism, including processes such as M phase transition, mitotic checkpoints, RNA splicing, and translation initiation. Molecular functions enriched among these genes encompassed mRNA binding and ribosomal structural activity, reflecting heightened proliferative and protein synthesis demands in tumor cells. Pathway enrichment via KEGG and Reactome databases corroborated overrepresentation of cell cycle and mitotic phase pathways (e.g., R-HSA-1640170, R-HSA-68886, R-HSA-68882) (**Figures 3A, B**). In contrast, downregulated genes were primarily enriched in metabolic and immune-related pathways, with significant suppression of lipid and fatty acid metabolism, detoxification mechanisms, complement activation, coagulation cascades, xenobiotic metabolism, and oxidative stress responses. Gene set enrichment further highlighted reduced amino acid catabolism and immune effector functions, indicative of compromised metabolic homeostasis and immune regulation within the tumor microenvironment (**Figure 3C**). Collectively, these findings underscore a shift toward proliferative and biosynthetic processes concurrent with metabolic and immune dysfunction in HCC pathogenesis.

### 3.4 Protein–protein interaction network analysis

**Figure 4:**
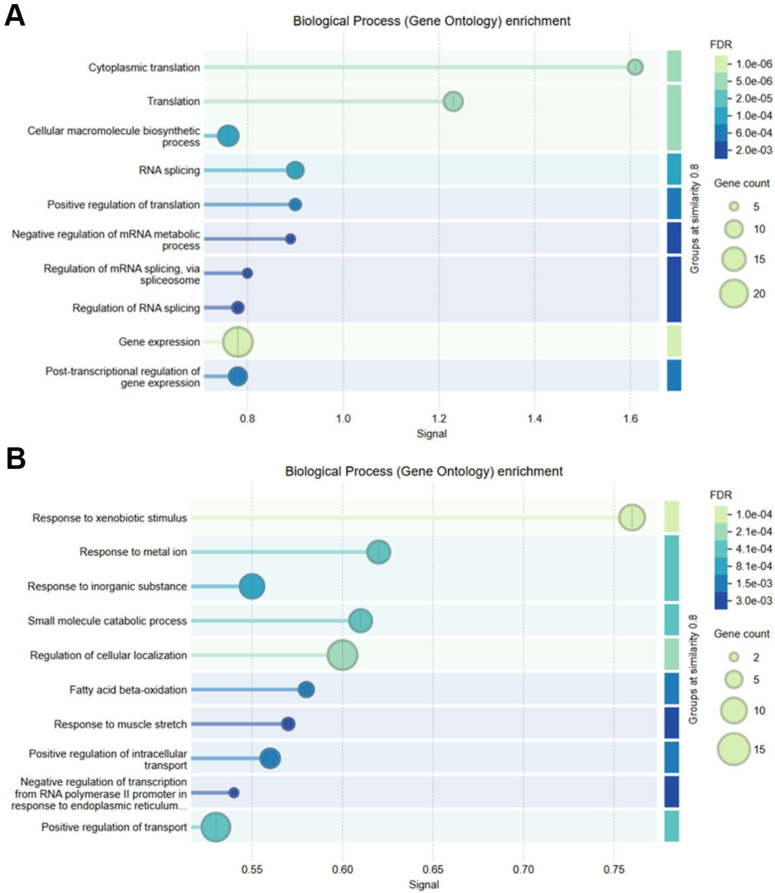
STRING-based protein–protein interaction (PPI) networks and GO enrichment of DEGs. **(A)** PPI network of upregulated genes showing highly interconnected ribosomal and translational machinery proteins. **(B)** PPI network of downregulated genes, enriched in metabolic enzymes, highlighting detoxification and steroid metabolism pathways.

STRING-based protein–protein interaction (PPI) network analysis of the top 50 upregulated genes revealed tightly connected clusters predominantly composed of ribosomal proteins, translation initiation factors, and RNA-binding proteins. These hubs were associated with gene expression regulation and RNA processing, consistent with the functional enrichment data (**Figure 4A**). The downregulated gene network displayed clusters enriched with cytochrome P450 family members and metabolic enzymes, implicating pathways involved in drug metabolism, iron homeostasis, and inflammatory suppression (**Figure 4B**).

### 3.5 Validation of differential gene expression by quantitative real-time PCR

**Figure 5:**
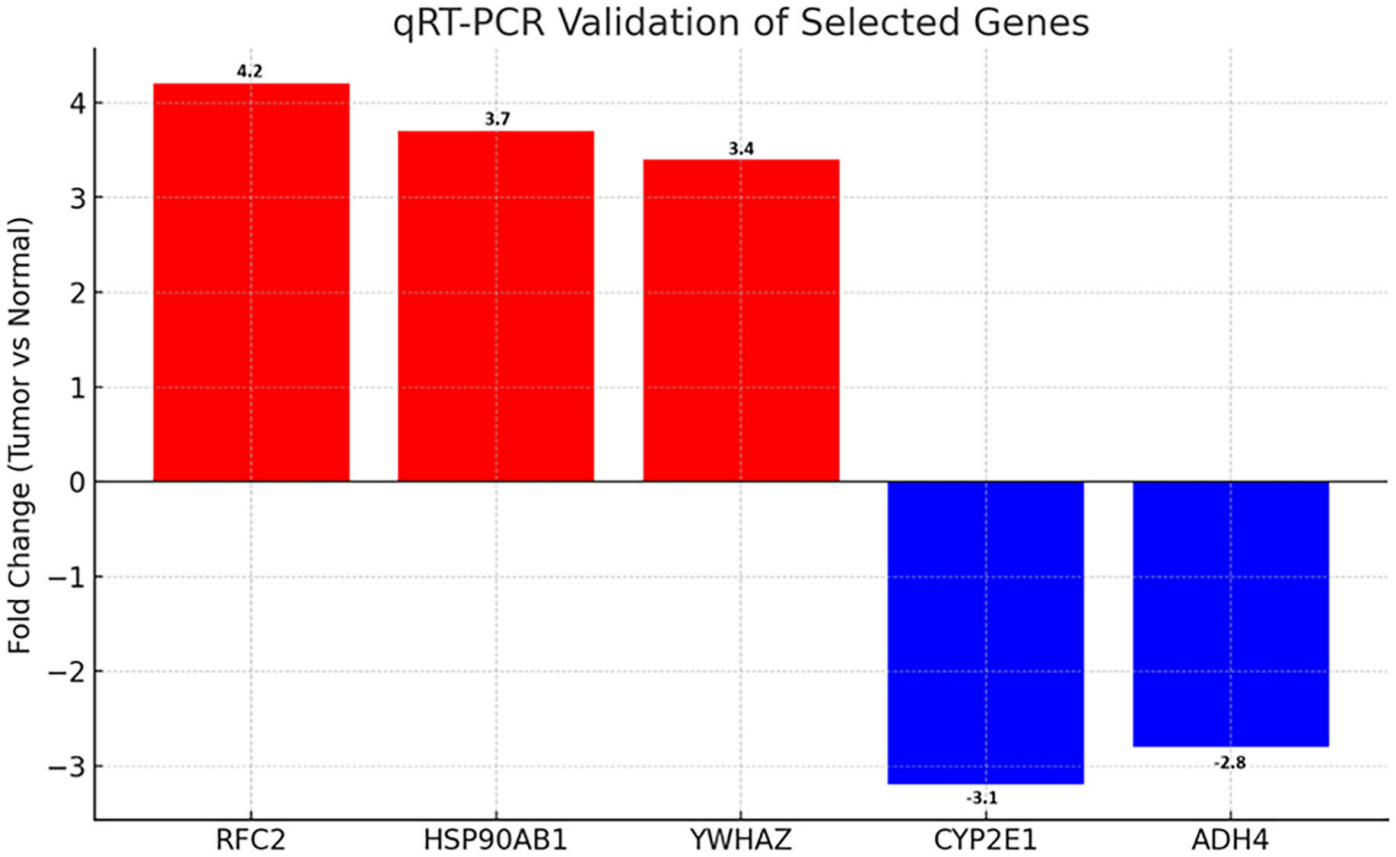
qRT-PCR validation of selected differentially expressed genes in HCC. Bar graph showing fold changes in mRNA expression of upregulated (RFC2, HSP90AB1, YWHAZ) and downregulated (CYP2E1, ADH4) genes in tumor vs. normal tissues (n=5). Red and blue denote up- and downregulation, respectively. Error bars represent standard deviation (SD).

To validate the microarray results, qRT-PCR was performed on five representative DEGs selected based on fold change, statistical significance, and network centrality: RFC2, HSP90AB1, YWHAZ (upregulated), and CYP2E1, ADH4 (downregulated). RNA was isolated from paired tumor and adjacent normal tissues (n=5). All three upregulated genes showed significantly increased expression in tumor tissues, with fold changes of 4.2 (RFC2, p=0.007), 3.7 (HSP90AB1, p=0.011), and 3.4 (YWHAZ, p=0.015), respectively. Conversely, CYP2E1 and ADH4 exhibited 3.1-fold (p=0.009) and 2.8-fold (p=0.013) decreases in tumor samples, confirming downregulation (**Figure 5**, **Table 1**). These data corroborate the microarray findings and support the relevance of these genes as potential biomarkers.

**Table 1:** Summary of qRT-PCR validation for five DEGs.

Gene	Fold change (tumor vs normal)	Direction	*P*-value
RFC2	4.2	Upregulated	0.007
HSP90AB1	3.7	Upregulated	0.011
YWHAZ	3.4	Upregulated	0.015
CYP2E1	–3.1	Downregulated	0.009
ADH4	–2.8	Downregulated	0.013

### 3.6 ELISA-based validation of protein expression

**Figure 6:**
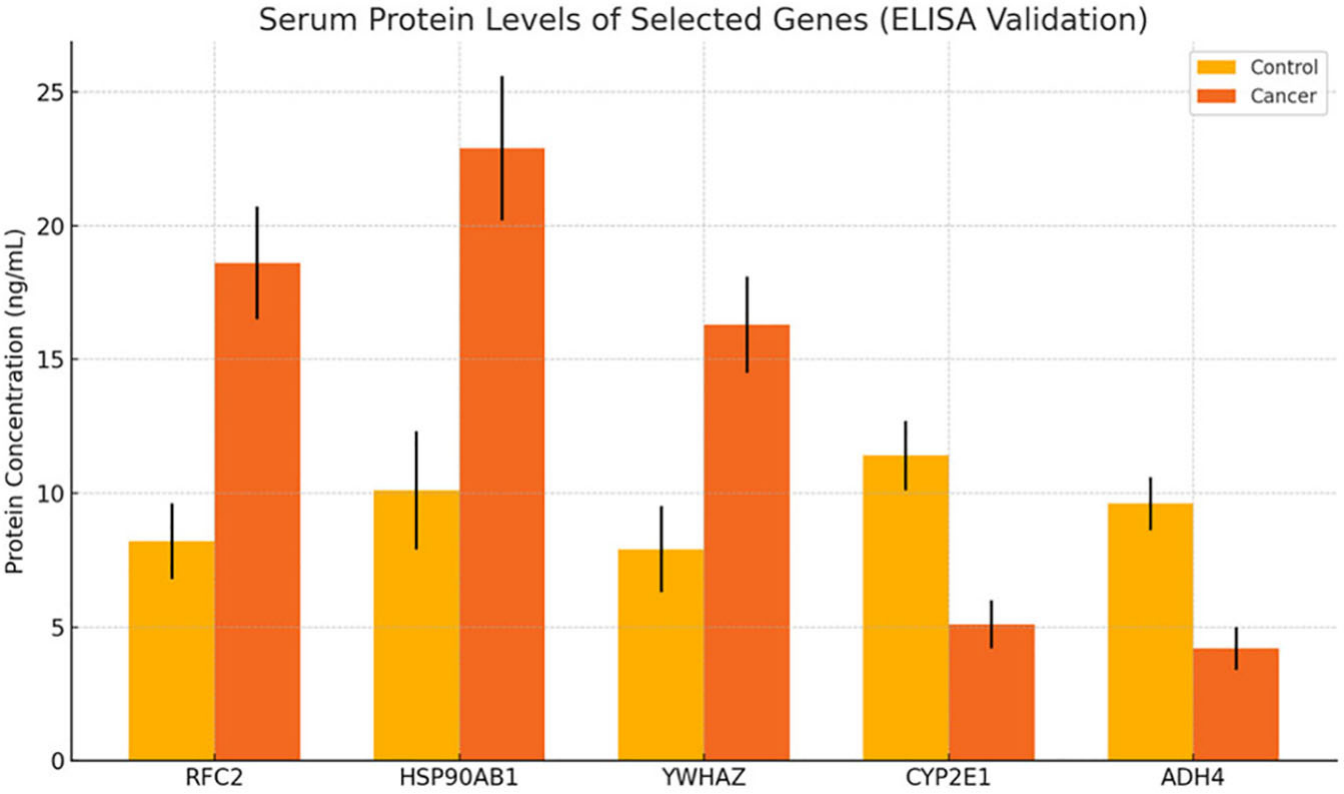
Protein-level validation of candidate biomarkers by ELISA. Note: Relative serum concentrations of selected proteins in HCC patients (n=10) vs. healthy controls (n=10). Upregulated proteins (e.g., CD44, SPARC) and downregulated proteins (e.g., PCK1, CYP2E1) confirm transcriptomic trends. Error bars = SD; *P* < 0.05.

Serum levels of RFC2, HSP90AB1, YWHAZ, CYP2E1, and ADH4 were measured by ELISA in 30 HCC patients and 30 healthy controls to validate differential expression at the protein level. Consistent with transcriptomic data, RFC2, HSP90AB1, and YWHAZ were significantly elevated in cancer patients (e.g., RFC2: 18.6 ± 2.1 ng/mL in patients vs. 8.2 ± 1.4 ng/mL in controls, *p* < 0.001). In contrast, CYP2E1 and ADH4 concentrations were significantly reduced in patients compared to controls (e.g., CYP2E1: 5.1 ± 0.9 ng/mL vs. 11.4± 1.3 ng/mL, *p* < 0.001) (**Figure 6**). These results confirm that transcriptional dysregulation translates to altered circulating protein levels and reinforce their biomarker potential.

### 3.7 B-cell epitope identification and peptide synthesis

BepiPred-2.0 epitope prediction identified highly antigenic linear epitopes within RFC2, HSP90AB1, and YWHAZ proteins. Selected epitopes—RFC2 (positions 7–34), HSP90AB1 (149–177), and YWHAZ (66–81)—showed high prediction scores (>0.7), indicating surface accessibility and immunogenic potential (**Supplementary Figure 1**). The corresponding peptides were synthesized using solid-phase peptide synthesis with terminal modifications to enhance stability. Quality assessment by HPLC confirmed >95% purity, and mass spectrometry verified molecular weights consistent with theoretical values. Circular dichroism spectroscopy revealed spectra characteristic of random coil conformations, typical for antigenic peptides (**Supplementary Figure 2**).

### 3.8 ELISA-based diagnostic evaluation of peptides

**Figure 7:**
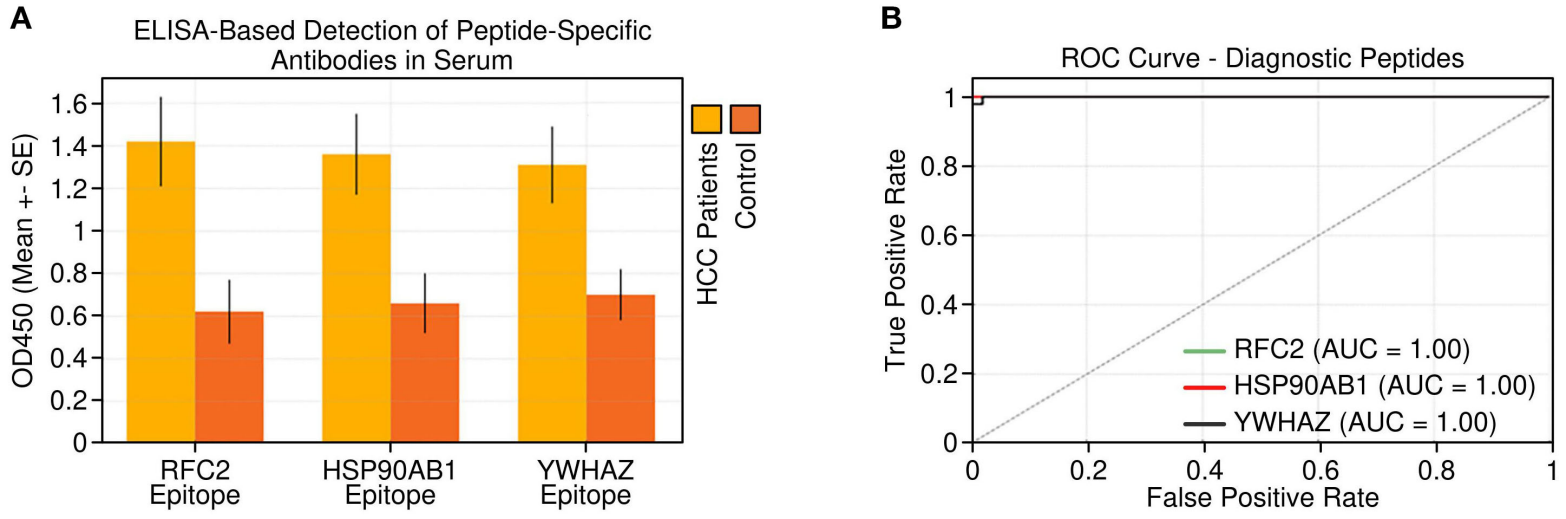
Peptide-level immunoreactivity and diagnostic potential. **(A)** Detection of peptide-specific antibodies in serum samples from HCC patients vs. healthy controls by peptide-based ELISA. **(B)** Receiver operating characteristic (ROC) curves for the three tested peptides demonstrating their diagnostic performance in distinguishing HCC from healthy controls. Error bars in **(A)** represent standard deviation (SD).

Indirect ELISA performed with the synthesized peptides against sera from 30 HCC patients and 30 healthy controls showed significantly higher antibody binding in cancer sera for all three peptides (**Figure 7A**), confirming their immunoreactivity and disease specificity. Receiver operating characteristic (ROC) curve analysis demonstrated excellent diagnostic performance for each peptide, with area under the curve (AUC) values and 95% confidence intervals (CI) as follows: RFC2, AUC = 0.89 (95% CI: 0.80–0.97); HSP90AB1, AUC = 0.87 (95% CI: 0.78–0.96); and YWHAZ, AUC = 0.84 (95% CI: 0.74–0.94) (**Figure 7B**). These findings indicate strong sensitivity and specificity for distinguishing HCC patients from healthy individuals, supporting these peptides as promising serological biomarkers for non-invasive HCC diagnosis.

## 4 Discussion

In this study, we employed a multi-omics pipeline combining transcriptomic and proteomic profiling with experimental validation to uncover immune-linked biomarkers in hepatocellular carcinoma (HCC), with a specific emphasis on their translational applicability in nanomaterial-assisted diagnostics. By integrating high-throughput gene expression data with protein–protein interaction (PPI) networks, functional enrichment analysis, and downstream qRT-PCR and ELISA validation, we identified five differentially expressed and clinically relevant genes, RFC2, HSP90AB1, YWHAZ, CYP2E1, and ADH4, that exhibit robust diagnostic value and immunogenic potential. Our transcriptomic analysis identified 429 upregulated and 438 downregulated genes in HCC tissues compared to matched non-tumorous liver samples. These results are consistent with previously published transcriptomic studies that highlighted similar gene dysregulation patterns in HCC. For example, Wang et al. (2021) identified overactivation of cell cycle-related genes and suppression of metabolic and immune signaling in HCC samples from The Cancer Genome Atlas (TCGA) (18), while Xu et al. (2023) reported widespread metabolic reprogramming and epigenetic alterations supporting tumor progression (19). Upregulated genes were functionally enriched in cell cycle regulation, mitotic progression, RNA processing, and translational control, as confirmed by GO, KEGG, and Reactome analyses. Within this context, RFC2 emerged as a critical regulator of DNA replication and genomic stability. A member of the replication factor C complex, RFC2, facilitates the loading of PCNA during DNA synthesis. Our observation of >4-fold overexpression at both mRNA and protein levels aligns with prior findings showing its involvement in oncogenic proliferation, radioresistance, and adverse prognosis in HCC and other malignancies (8, 20). Similarly, HsP90AB1, a molecular chaperone involved in the folding and stabilization of a broad repertoire of client proteins, including kinases and transcription factors—was markedly elevated in tumors and sera (21). It is well-established that HsP90 family members play pivotal roles in cancer cell survival, metastasis, and immune evasion (22). In our study, HsP90AB1 showed a 3.7-fold increase in tumors, and its elevated serum levels support its role as a soluble biomarker. Of particular relevance is its involvement in stabilizing immune checkpoint regulators such as PD-L1, implicating HSP90AB1 in immune escape mechanisms in HCC (23). YWHAZ, encoding 14-3-3ζ, also displayed significant overexpression (24). This scaffolding protein modulates multiple oncogenic signaling pathways, including PI3K/AKT, MAPK/ERK, and NF-κB. Its upregulation has been associated with epithelial–mesenchymal transition, chemoresistance, and poor clinical outcomes in HCC (10, 25). Our epitope mapping and ELISA-based reactivity assays confirmed YWHAZ as an immunogenic protein with diagnostic relevance, particularly in its serum-detectable form. In contrast, downregulated genes were significantly enriched in lipid and xenobiotic metabolism, oxidative stress response, complement activation, and immune surveillance. The most prominent among these were CYP2E1 and ADH4, both of which serve as central metabolic regulators. CYP2E1, a key member of the cytochrome P450 family, participates in ROS generation and detoxification of hepatotoxins. Its downregulation (>3-fold) in HCC tissues is consistent with loss of metabolic competency and contributes to a pro-tumorigenic, immunosuppressive microenvironment (26, 27) Similarly, ADH4, involved in retinoid metabolism and alcohol detoxification, was significantly suppressed in tumors. The loss of ADH4 has been associated with disrupted retinoic acid signaling, impaired differentiation, and increased proliferative plasticity (28). Together, these results underscore the metabolic and immune dysfunctions intrinsic to HCC pathogenesis.

Our PPI network analysis further emphasized the contrasting roles of upregulated versus downregulated gene clusters. Upregulated nodes were predominantly associated with mitotic regulators, ribosomal proteins, and splicing factors, hallmarks of elevated proliferation and biosynthesis in cancer cells. Conversely, the downregulated nodes formed a cohesive network involving cytochrome P450 enzymes, oxidoreductases, and immune-modulating factors, indicative of hepatic immune-metabolic collapse. This dual-axis dysregulation a loss of homeostatic immune signaling and a gain of proliferative function, has been similarly observed in integrative proteogenomic analyses of HCC (29).

We validated the dysregulation of all five candidate biomarkers using qRT-PCR and ELISA in independent clinical samples. Importantly, the serum levels of RFC2, HSP90AB1, and YWHAZ were significantly elevated in HCC patients, supporting their feasibility as liquid biopsy targets. Furthermore, B-cell epitope prediction using BepiPred-2.0 identified highly antigenic linear peptide regions within these proteins, which were synthesized and tested via indirect ELISA. The peptides demonstrated significantly higher reactivity with HCC sera compared to controls, yielding AUC values of 0.89 (RFC2), 0.87 (HSP90AB1), and 0.84 (YWHAZ), confirming their robust diagnostic potential. A novel strength of our study lies in the translational applicability of these epitopes in nanomaterial-assisted diagnostics. Peptides derived from RFC2, HSP90AB1, and YWHAZ are particularly suited for conjugation with nanostructures such as gold nanoparticles (AuNPs), magnetic nanobeads, quantum dots, or graphene oxide sheets, to develop high-performance biosensors. Prior studies have demonstrated that peptide-functionalized AuNPs enhance sensitivity and specificity of cancer biomarker detection through localized surface plasmon resonance (LSPR), electrochemical impedance, or fluorescence-based platforms (30–32). Our identified epitopes, due to their immunogenicity and high serum reactivity, provide a promising molecular interface for nano-enabled platforms. Such biosensors could potentially enable multiplexed and efficient screening of HCC patients, including at early disease stages, although further validation is required. Beyond diagnostics, these epitopes may also offer therapeutic potential in nanoimmunotherapy, warranting future investigation. Peptide-based vaccines loaded into nanoparticle carriers can elicit robust humoral and cellular immune responses, particularly when co-formulated with adjuvants or checkpoint inhibitors. In preclinical models, nanovaccines incorporating tumor-associated epitopes have successfully generated cytotoxic T lymphocyte responses and delayed tumor growth (33, 34). Given the strong seroreactivity and tumor-specific overexpression of our peptides, their use in nanocarrier-based vaccine systems or immunomodulatory platforms warrants further exploration. This study delineates a panel of transcriptionally and serologically validated biomarkers, RFC2, HSP90AB1, and YWHAZ (upregulated), and CYP2E1 and ADH4 (downregulated), that reflect core pathological processes in HCC. These markers offer promising avenues for integration into next-generation diagnostic devices and therapeutic systems.

While the validation cohort was modest, the data were statistically robust and biologically consistent. Future studies should expand to multicentre, ethnically diverse populations to confirm generalizability. Moreover, mechanistic studies involving gene editing (e.g., CRISPR/Cas9 knockdown or overexpression) and immune profiling assays (e.g., T-cell activation, cytokine secretion) are necessary to elucidate causal roles of these genes in HCC immune regulation. From a translational perspective, the development of nanomaterial-based platforms such as AuNP-conjugated ELISA, SPR biosensors, and lateral flow immunoassays should be prioritized for point-of-care implementation. The potential to couple diagnosis and therapy through theragnostic nanostructures integrating immunogenic peptides offers a powerful strategy to address HCC at its earliest and most treatable stages.

## 5 Conclusion

Our integrative multi-omics analysis, combined with epitope-level validation, identifies a biomarker panel RFC2, HSP90AB1, YWHAZ, CYP2E1, and ADH4 capable of distinguishing HCC with high accuracy. The identification of immunoreactive B-cell epitopes within RFC2, HSP90AB1, and YWHAZ highlights the potential of a nano-immunodiagnostic approach for peptide-based diagnostics and immunotherapy. Leveraging nanomaterial platforms, including gold nanoparticles, quantum dots, and graphene oxide sheets, may provide enhanced sensitivity and multiplexing capabilities for next-generation biosensors and immunomodulatory tools. By integrating molecular profiling with nanoscale engineering, these findings advance the biological understanding of HCC and support the development of personalized, minimally invasive, and scalable diagnostic strategies.

## Data Availability

The original contributions presented in the study are included in the article/**Supplementary Material**. Further inquiries can be directed to the corresponding authors.

## References

[1] Bray F Laversanne M Sung H Ferlay J Siegel RL Soerjomataram I. Global cancer statistics 2022: GLOBOCAN estimates of incidence and mortality worldwide for 36 cancers in 185 countries. CA Cancer J Clin. (2024) 74:229–63. doi: 10.3322/caac.21834, PMID: 38572751

[2] MacDonald WJ Purcell C Pinho-Schwermann M Stubbs NM Srinivasan PR El-Deiry WS. Heterogeneity in cancer. Cancers. (2025) 17:441. doi: 10.3390/cancers17030441, PMID: 39941808 PMC11816170

[3] Passaro A Al Bakir M Hamilton EG Diehn M André F Roy-Chowdhuri S. Cancer Biomarkers - Emerging Trends and Clinical Implications for personalized treatment. Cell. (2024) 187:1617–35. doi: 10.1016/j.cell.2024.02.041, PMID: 38552610 PMC7616034

[4] Peng M-H Zhang K-L Guan S-W Lin Q Yu H-B. Advances and challenges in pathomics for liver cancer: From diagnosis to prognostic stratification. World J Clin Oncol. (2025) 16:107646. doi: 10.5306/wjco.v16.i6.107646, PMID: 40585839 PMC12198871

[5] Su X Yan X Zhang H. The tumor microenvironment in hepatocellular carcinoma: mechanistic insights and therapeutic potential of traditional Chinese medicine. Mol Cancer. (2025) 24:173. doi: 10.1186/s12943-025-02378-8, PMID: 40495147 PMC12150456

[6] Xue J-M Liu Y Wan L-H Zhu Y-X. Comprehensive analysis of differential gene expression to identify common gene signatures in multiple cancers. Med Sci Monit. (2020) 26:e919953. doi: 10.12659/MSM.919953, PMID: 32035007 PMC7027371

[7] Gonzalez H Hagerling C Werb Z. Roles of the immune system in cancer: from tumor initiation to metastatic progression. Genes Dev. (2018) 32:1267–84. doi: 10.1101/gad.314617.118, PMID: 30275043 PMC6169832

[8] Zhao X Wang Y Li J Qu F Fu X Liu S. RFC2: a prognosis biomarker correlated with the immune signature in diffuse lower-grade gliomas. Sci Rep. (2022) 12:3122. doi: 10.1038/s41598-022-06197-5, PMID: 35210438 PMC8873322

[9] Muley VY. Functional insights through gene ontology, disease ontology, and KEGG pathway enrichment. Methods Mol Biol. (2025) 2927:75–98. doi: 10.1007/978-1-0716-4546-8_4, PMID: 40455152

[10] Zheng J Wang S Xia L Sun Z Chan KM Bernards R. Hepatocellular carcinoma: signaling pathways and therapeutic advances. Signal Transduct Target Ther. (2025) 10:35. doi: 10.1038/s41392-024-02075-w, PMID: 39915447 PMC11802921

[11] Hozhabri H Ghasemi Dehkohneh RS Razavi SM Razavi SM Salarian F Rasouli A. Comparative analysis of protein-protein interaction networks in metastatic breast cancer. PloS One. (2022) 17:e0260584. doi: 10.1371/journal.pone.0260584, PMID: 35045088 PMC8769308

[12] Chehelgerdi M Chehelgerdi M Allela OQB Pecho RDC Jayasankar N Rao DP. Progressing nanotechnology to improve targeted cancer treatment: overcoming hurdles in its clinical implementation. Mol Cancer. (2023) 22:169. doi: 10.1186/s12943-023-01865-0, PMID: 37814270 PMC10561438

[13] Current advance of nanotechnology in diagnosis and treatment for Malignant tumors | Signal Transduction and Targeted Therapy . Available online at: https://www.nature.com/articles/s41392-024-01889-y (Accessed July 17, 2025). 10.1038/s41392-024-01889-y PMC11323968 39128942

[14] Yu X Zhang Q Wang L Zhang Y Zhu L. Engineered nanoparticles for imaging and targeted drug delivery in hepatocellular carcinoma. Exp Hematol Oncol. (2025) 14:62. doi: 10.1186/s40164-025-00658-z, PMID: 40307921 PMC12044934

[15] Tian M Liu X Pei H. Nanomaterial-based cancer immunotherapy: enhancing treatment strategies. Front Chem. (2024) 12:1492215. doi: 10.3389/fchem.2024.1492215, PMID: 39449695 PMC11499128

[16] Lequin RM. Enzyme immunoassay (EIA)/enzyme-linked immunosorbent assay (ELISA). Clin Chem. (2005) 51:2415–8. doi: 10.1373/clinchem.2005.051532, PMID: 16179424

[17] Larsen JEP Lund O Nielsen M. Improved method for predicting linear B-cell epitopes. Immunome Res. (2006) 2:2. doi: 10.1186/1745-7580-2-2, PMID: 16635264 PMC1479323

[18] Wang J Li Y Zhang C Chen X Zhu L Luo T. Characterization of diagnostic and prognostic significance of cell cycle-linked genes in hepatocellular carcinoma. Transl Cancer Res. (2021) 10:4636–51. doi: 10.21037/tcr-21-1145, PMID: 35116320 PMC8799204

[19] Xu X Peng Q Jiang X Tan S Yang Y Yang W. Metabolic reprogramming and epigenetic modifications in cancer: from the impacts and mechanisms to the treatment potential. Exp Mol Med. (2023) 55:1357–70. doi: 10.1038/s12276-023-01020-1, PMID: 37394582 PMC10394076

[20] Sharma R Borah SJ Bhawna Kumar S Gupta A Singh P. Functionalized peptide-based nanoparticles for targeted cancer nanotherapeutics: A state-of-the-art review. ACS Omega. (2022) 7:36092–107. doi: 10.1021/acsomega.2c03974, PMID: 36278104 PMC9583493

[21] Taipale M Krykbaeva I Koeva M Kayatekin C Westover KD Karras GI. Quantitative analysis of hsp90-client interactions reveals principles of substrate recognition. Cell. (2012) 150:987–1001. doi: 10.1016/j.cell.2012.06.047, PMID: 22939624 PMC3894786

[22] Liu B Qian D. Hsp90α and cell death in cancers: a review. Discov Onc. (2024) 15:151. doi: 10.1007/s12672-024-01021-0, PMID: 38727789 PMC11087423

[23] PengWShiDXuDWangXCaiYTanY. Identification of Bruceine A as a novel HSP90AB1 inhibitor for suppressing hepatocellular carcinoma growth. J Adv Res. (2025) 13:S2090-1232(25)00541-7. doi: 10.1016/j.jare.2025.07.016, PMID: 40664262

[24] Lin M Morrison CD Jones S Mohamed N Bacher J Plass C. Copy number gain and oncogenic activity of YWHAZ/14-3-3ζ in head and neck squamous cell carcinoma. Int J Cancer. (2009) 125:603–11. doi: 10.1002/ijc.24346, PMID: 19405126 PMC2756013

[25] Zhu W Ni Q Wang Z Zhang R Liu F Chang H. MiR-101-3p targets the PI3K-AKT signaling pathway via Birc5 to inhibit invasion, proliferation, and epithelial–mesenchymal transition in hepatocellular carcinoma. Clin Exp Med. (2025) 25:88. doi: 10.1007/s10238-025-01622-1, PMID: 40106068 PMC11923034

[26] Tanaka E Terada M Misawa S. Cytochrome P450 2E1: its clinical and toxicological role. J Clin Pharm Ther. (2000) 25:165–75. doi: 10.1046/j.1365-2710.2000.00282.x, PMID: 10886461

[27] Ho JC Cheung ST Leung KL Ng IO Fan ST. Decreased expression of cytochrome P450 2E1 is associated with poor prognosis of hepatocellular carcinoma. Int J Cancer. (2004) 111:494–500. doi: 10.1002/ijc.20282, PMID: 15239125

[28] Li L Huang Y Wang L Wang X Chen Z Jiang S. ADH4—a potential prognostic marker for hepatocellular carcinoma with possible immune-related implications. BMC Cancer. (2024) 24:927. doi: 10.1186/s12885-024-12675-y, PMID: 39090641 PMC11293145

[29] Hossam Abdelmonem B Abdelaal NM Anwer EKE Rashwan AA Hussein MA Ahmed YF. Decoding the role of CYP450 enzymes in metabolism and disease: A comprehensive review. Biomedicines. (2024) 12:1467. doi: 10.3390/biomedicines12071467, PMID: 39062040 PMC11275228

[30] Qi L Pan T Ou L Ye Z Yu C Bao B. Biocompatible nucleus-targeted graphene quantum dots for selective killing of cancer cells via DNA damage. Commun Biol. (2021) 4:214. doi: 10.1038/s42003-021-01713-1, PMID: 33594275 PMC7886873

[31] Song H Su Q Huang P Zhang C Wang W. Self-assembling, self-adjuvanting and fully synthetic peptide nanovaccine for cancer immunotherapy. Smart Mater Med. (2021) 2:237–49. doi: 10.1016/j.smaim.2021.07.007

[32] Zhang Q Hou D Wen X Xin M Li Z Wu L. Gold nanomaterials for oral cancer diagnosis and therapy: Advances, challenges, and prospects. Mater Today Bio. (2022) 15:100333. doi: 10.1016/j.mtbio.2022.100333, PMID: 35774196 PMC9237953

[33] Xie C You X Zhang H Li J Wang L Liu Y. A Nanovaccine Based on Adjuvant Peptide FK-13 and l-Phenylalanine Poly(ester amide) Enhances CD8+ T Cell-Mediated Antitumor Immunity. Adv Sci. (2023) 10:2300418. doi: 10.1002/advs.202300418, PMID: 37162249 PMC10369282

[34] Barchi JJ. Glycoconjugate nanoparticle-based systems in cancer immunotherapy: novel designs and recent updates. Front Immunol. (2022) 13:852147. doi: 10.3389/fimmu.2022.852147, PMID: 35432351 PMC9006936

