# Supplementary material for: Nanomaterial-assisted immunodiagnostic profiling and therapeutic targeting of hepatocellular carcinoma: from molecular biomarkers to clinical applications
Source: Front Immunol. 2025 Oct 14;16:1668630. doi: 10.3389/fimmu.2025.1668630 (PMC12558944; doi:10.3389/fimmu.2025.1668630)
Supplement: Supplementary file 1 [file DataSheet1.docx]

**Supplementary Data**


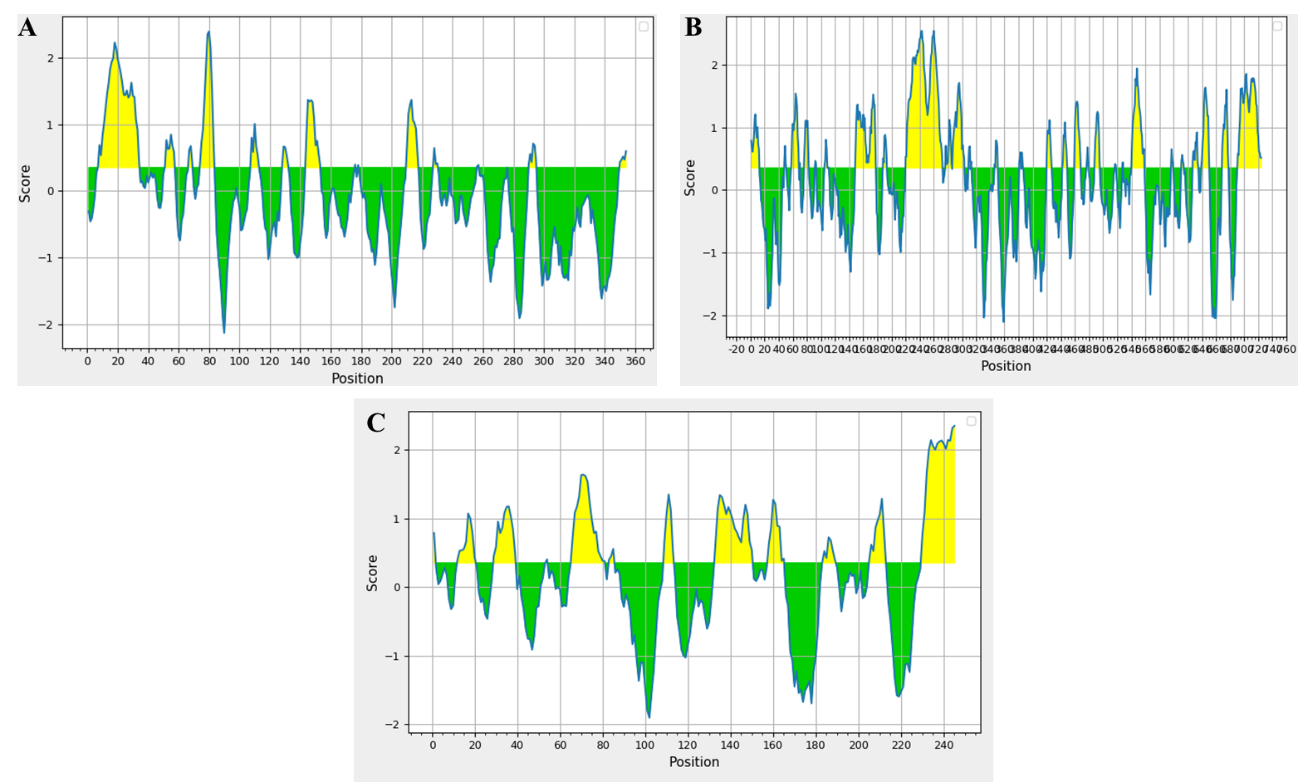
 **Supplementary figure 1:** B cell epitopes prediction for proteins: RFC2 (A), HSP90AB1 (B), and YWHAZ (C) by Bepipred server


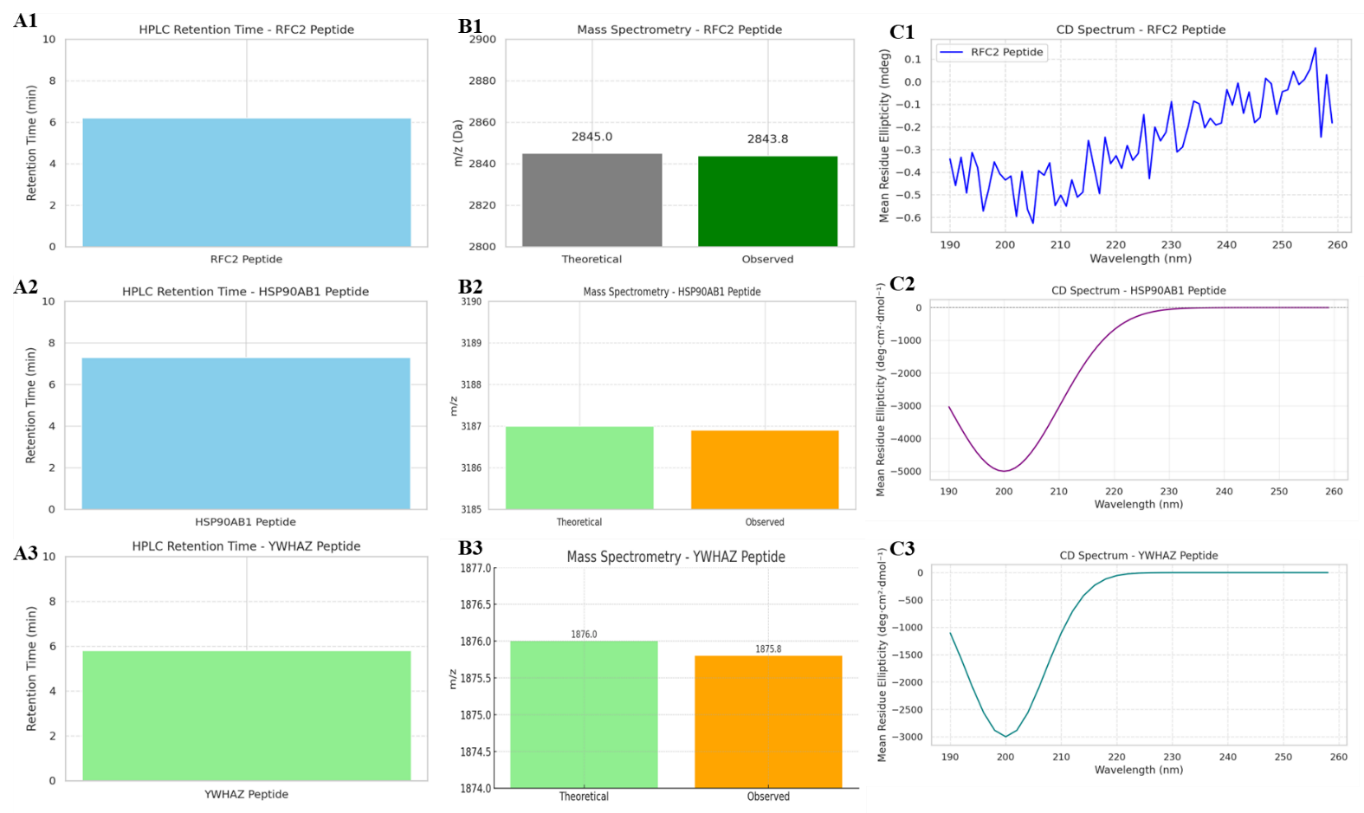


**Supplementary figure 2:** A1-A3: HPLC of all 3 peptides, B1-B3: MS of all 3 peptides, C1-C3: CD spectrum of all three peptides.
